# Associations of Socioeconomic Status Inequity with Incident Age-related Macular Degeneration in Middle-Aged and Elderly Population

**DOI:** 10.34133/hds.0148

**Published:** 2024-07-01

**Authors:** Yanlin Qu, Guanran Zhang, Zhenyu Wu, Huihuan Luo, Renjie Chen, Huixun Jia, Xiaodong Sun

**Affiliations:** ^1^ Department of Ophthalmology, Shanghai General Hospital, Shanghai Jiao Tong University School of Medicine, Shanghai, China.; ^2^ Shanghai Key Laboratory of Ocular Fundus Diseases, Shanghai, China.; ^3^ National Clinical Research Center for Eye Diseases, Shanghai, China.; ^4^ School of Public Health, Fudan University, Shanghai, China.

## Abstract

**Background:** The relationship between socioeconomic status (SES) inequity and incident age-related macular degeneration (AMD) remains unclear. We aim to investigate whether low SES increases the risk of AMD and to explore the effect of a healthy lifestyle on this association. **Methods:** This prospective cohort study included 316,663 UK Biobank individuals. SES inequity was identified via latent class analysis using education, household income, and occupational status. Healthy lifestyle score was calculated based on smoking, alcohol drinking, and physical activity (PA). Incident AMD was defined according to diagnosis records. Cox proportional hazards models were used to evaluate the relationship of low SES and AMD. Interrelationships of healthy lifestyle score on SES–AMD association were explored, including modification, mediation, and joint effects. **Results:** During the average 12.2 years of follow-up, 6,355 AMD cases were diagnosed. Participants with medium SES (hazard ratio: 1.10 [95% confidence interval (CI) 1.01 to 1.21]) and low SES (hazard ratio: 1.22 [95% CI 1.11 to 1.34]) had an increased risk of incident AMD compared to participants with high SES. PA significantly affected this association. Moreover, the association between low SES and AMD was significantly mediated (11.3%, 95% CI: 6.56 to 23.0) by smoking. Similarly, alcohol drinking suppressed (9.59%, 95% CI: 4.00 to 23.2) the association between high SES and AMD. Besides, a significant joint effect of SES and healthy lifestyle score was found. **Conclusions:** We provide further evidence for the relationship of socioeconomic inequity, healthy lifestyle, and incident AMD. Future public health strategies should aim to reduce socioeconomic inequity to prevent AMD.

## Introduction

Rapid socioeconomic development has led to the improved quality of life over the recent decades. However, this progress has concurrently exacerbated social inequities [1,2]. These disparities, stemming from socioeconomic differences, become even more pronounced during the COVID-19 pandemic, especially in the UK [3]. The hazard of socioeconomic status (SES) inequity on diseases has been explored extensively, particularly in age-related diseases [4–7]. Previous studies demonstrated that individuals with a lower SES had an elevated risk of dementia [6,7], cardiovascular disease [5], and cancer [4]. Yet, the impact of SES inequity on age-related macular degeneration (AMD), the leading cause of blindness in developed countries [8], remains unclear [9–18]. Previous studies hinted toward an association between SES inequity and AMD [9–18]; however, these studies were limited to the fact that they were either cross-sectional [14–16] or with small sample size [9,10], lacking sufficient evidence and statistical power. To promote quality of life in the elderly population, it is imperative to evaluate the association of SES with incident AMD and demonstrate the impacts of SES inequity on ocular health.

In addition to the unclear relationship, previous studies usually defined personal SES via single dimension, such as education, occupation, income, or insurance [9–16]. These definitions may not adequately capture the comprehensive essence of SES [19] and may overlook the interrelation of SES factors. Therefore, it is necessary to determine an overall SES variable containing diverse aspects from a population-based cohort. Furthermore, the underlying mechanisms on how SES relates to AMD remain unclear. Healthy lifestyle behaviors may play important roles, as they are commonly recognized as mediators of SES–diseases association [20], and their modification and joint effects with SES on health outcomes have been established in recent studies [21]. Nevertheless, these complex impacts of lifestyle behaviors on the association between SES and AMD have yet to be explored.

To elucidate the above-mentioned research gaps, our study firstly performed an association analysis to examine whether SES inequity would contribute to an increased risk of AMD using the population-based data from the UK Biobank. Then we further investigated the complex impacts of healthy lifestyle on SES–AMD association, aiming to provide targeted strategies for AMD prevention.

## Methods

### Study population

As a population-based prospective cohort, the UK Biobank enrolled over 500,000 individuals ranging from 40 to 69 years old, gathered from 22 assessment centers situated in England, Wales, and Scotland during the period from 2006 to 2010 [22]. In this study, individuals with missing information on educational level (*N* = 10,132), household income (*N* = 68,885), and employment status (*N* = 2,150) were excluded. Then, 421,229 participants remained to identify the overall SES groups. Subsequently, we excluded those with AMD at baseline (*N* = 532) and with age <50 years (*N* = 104,034). Finally, 316,663 individuals were included in the association analyses. The details were shown in Fig. S1.

The protocols of the UK Biobank have been approved by the National Information Governance Board for Health and Social Care and National Health Service North West Multicenter Research Ethics Committee. All participants provided informed written consent at their first visit to the assessment center.

### Assessment of SES

We assessed individual SES from 3 aspects, including educational level, household income, and employment status as proposed. Health insurance coverage was not considered as a component of SES due to National Health Service, a publicly funded healthcare system in the UK [21].

In the UK Biobank, participants were asked their education qualifications, and the results were recorded as college or university degree; Advanced levels, Advanced Subsidiary levels, or equivalent; Ordinary levels, General Certificate of Secondary Educations, or equivalent; Certificate of Secondary Educations or equivalent; National Vocational Qualification, Higher National Diplomas, Higher National Certificates, or equivalent; other professional qualifications; none of the above; prefer not to answer. Education qualifications of participants who chose “none of the above” were defined as “equivalent or less than high school”, and those of participants who chose “prefer not to answer” were treated as missing values, respectively [21,23]. Total household income before tax was categorized according to the “*Data Field 738*” questionnaire of the UK Biobank, which was designed to recognize the trends, changes, and patterns of economic status in the UK. Individuals chose an option from <£18,000; £18,000-£30,999; £31,000-£51,999; £52,000-£100,000; >£100,000; do not know; prefer not to answer. Those choosing the last 2 options were excluded as missing data [21,23]. For employment status, because of the unclear rank order, we simply regrouped participants into 2 groups: employed (those reported in paid employment or self-employed, retired, doing unpaid or voluntary work, or being full or part time students) and unemployed (those reported others) [21].

Latent class analysis (LCA) model was used to identify the SES group via the 3 variables above. The details of method and process were described in Section S1. LCA is an unsupervised clustering method, by which the potential subgroups of SES could be defined considering education, income, and occupation simultaneously. In this study, 3 latent classes were identified, representing a high, medium, and low SES according to the item-response probabilities, respectively.

Furthermore, Townsend deprivation index (TDI) was also included as an area level SES, which considered household overcrowding, car ownership, owner occupation, and unemployment [24]. A higher TDI indicated lower area level SES.

### Outcome

The main outcome was the diagnosis of AMD, determined through a combination of primary and secondary codes from the International Classification of Diseases (ICD)-9 and ICD-10, specifically Code H35.3 in ICD-10 and Code 362.5 in ICD-9. All stages of AMD were encompassed without differentiation [8]. Participants were monitored from the recruitment date until the occurrence of the AMD diagnosis, death, loss to follow-up, or the end of the study period (November 2021), whichever transpired first.

### Assessment of healthy lifestyle score and covariates

In the current study, we selected 4 candidate lifestyle behaviors to construct a healthy lifestyle score according to previous studies [21,25], including smoking, alcohol drinking, physical activity (PA), and healthy diet.

We defined never smoking as the healthy control, while previous or current smoking was considered as a risk behavior. Based on the frequency and volume of the alcohol consumption, individuals were divided into normal drinker and heavy drinker. Heavy drinker was defined as daily consumption of more than 1 drink for women and more than 2 drinks for men (1 drink contains 8 g of ethanol), respectively [21]. In term of PA, the frequency and time were asked for individuals in the UK Biobank. Participants meeting one of the following were defined as regular PA [23]: (a) for the frequency, vigorous activity ≥1 day/week or moderate activity ≥5 days/week; (b) for the time, vigorous activity ≥75 min/week or moderate activity ≥150 min/week. The assessment of a healthy dietary pattern was based on the fulfillment of 4 criteria across 6 components, including higher consumption of fruit, vegetables, and fish, while lower consumption of processed or unprocessed meats, and whole grains [23].

For each behavior, a healthy behavior was assigned 1 while 0 for the unhealthy behavior. Except the diet pattern, smoking, alcohol drinking, and physical inactivity were all significantly associated with incident AMD (Section S2). Thus, we constructed a healthy lifestyle score through the summation of these 3 behaviors, with higher scores indicating healthier lifestyles.

A series of sociodemographic covariates were collected by the questionnaire in the UK Biobank at baseline, including age, sex (male/female), self-report race (whites/others), assessment centers, and body mass index (BMI). Additionally, self-reported history of hypertension, diabetes, and cancer was also extracted.

### Statistical analysis

The analytical framework consisted of 2 parts, including evaluating the SES–AMD association and exploring the complex impact of healthy lifestyle score on this association.

In the association analyses, Cox proportional hazard regression models were used to evaluate the relationship between SES group and incident AMD. Hazard ratios (HRs) and 95% confidence intervals (CIs) were reported. Based on the previous studies [25,26], we established 3 models: model 1 unadjusted; model 2 adjusted for baseline age and sex, race, assessment center, BMI, self-report histories of hypertension, diabetes, and cancer; and model 3 with additional adjustment for the healthy lifestyle score. Stratified analyses were conducted among age, race, and BMI to explore whether the magnitudes of SES were varied in different subgroups.

Subsequently, the role of healthy lifestyle score on SES–AMD association was explored, including modification, mediation, and joint effects. The modification effect between SES and healthy lifestyle score was estimated through adding multiplicative interaction term in Cox hazard regression model. The HR (95% CI) of the product term was the measure of interaction on the multiplicative scale, and *P* <0.05 indicated significant modification effect. Mediation analyses were constructed using “mediation” package in R (version 4.1.3) to examine whether the SES–AMD association was mediated by healthy lifestyle score. Before the mediation analyses, we determined whether lifestyle was a mediator by comparing the effect of SES on AMD between model 2 and model 3. Logistic regression models were used to estimate the detailed mediation effects for healthy lifestyle score. The proportion of mediation was reported, and *P* <0.05 indicated significant mediation effect. For joint effect, participants were divided into 9 groups according to their SES and healthy lifestyle score. Compared with those in the High-SES group with 2 to 3 healthy lifestyle scores, the HRs of incident AMD in other groups were estimated. In addition, *P* for trend <0.05 indicated significant joint effect. We also repeated these analyses for each lifestyle behaviors.

Moreover, we implemented several sensitivity analyses to examine the robustness of our findings. Firstly, we designed 2 scenarios to evaluate whether the results would be biased by different definitions of the healthy lifestyle score: (a) the healthy lifestyle score was calculated by the simple additive method. Thus, a weighted score was established through partial regression coefficient of each behavior to explain for varied associations between different behaviors and incident AMD; (b) the healthy lifestyle score was calculated by including diet pattern and BMI. Secondly, to examine whether the effect of personal level SES on incident AMD was independent of area level SES, we further included TDI in model 3. Thirdly, to control selection bias, we excluded those with prevalent hypertension, diabetes, and cancer at baseline because these subjects may present a higher risk of incident AMD than those without chronic diseases. Additionally, we used multiple imputation via chained equations to impute the missing data on SES factors to test the influence of missing variables. Fourthly, to avoid reverse causation bias, we excluded those with AMD cases occurring in the first 2 years of follow-up. Fifthly, we additionally adjusted blood pressure and total cholesterol in model 3. Finally, we reanalyzed the association between SES and incident AMD based on a competing risk model.

Baseline characteristics were described via mean (SD) and n (%), as appropriate. All analyses were preformed using R software (version 4.1.3). Hypothesis tests were 2-sided, and we considered *P* <0.05 as statistically significant.

## Results

### Population characteristics

A total of 316,663 participants (mean age 59.8 years, 48.1% males) were included in the current study, and their baseline characteristics categorized by AMD status were presented in Table 1. During 3,877,557 person-years of follow-up (mean: 12.2 years, minimum–maximum: 0.01 to 14.9 years), 6,355 (2.01%) AMD cases were diagnosed. AMD cases were older, less often male, and less regularly active, while consuming cigarettes more often. Additionally, they had a higher BMI, lower overall SES, lower education, and household income and were more frequently unemployed. Compared with healthy controls, incident AMD subjects accompanied a higher probability of a history of diseases, including hypertension, diabetes, and cancer (*P* < 0.001 for all). The comparison of participants included and excluded was depicted in Table S1. Compared with included participants, excluded people were younger, less often male, and with relative higher educational level and household income.

**Table 1.** Baseline characteristics of participants by incident age-related macular degeneration. Variables were described using mean (SD), and *n* (%), as appropriate.

Characteristics	Without AMD *N* = 310,308	With AMD *N* = 6,355	*P* value
Age, years	59.8 (5.4)	63.3 (4.6)	<0.001
Males, *n* (%)	149,760 (48.3)	2,623 (41.3)	<0.001
White ethnicity or race, *n* (%)	2,98551 (96.2)	6,084 (95.7)	0.115
SES, *n* (%)			
High	5,1056 (16.5)	583 (9.2)	
Medium	160,435 (51.7)	3,006 (47.3)	
Low	98,817 (31.8)	2,766 (43.5)	<0.001
Education, *n* (%)			
College or above	121,304 (39.1)	2,206 (34.7)	
High school or equivalent	132,444 (42.7)	2,610 (41.1)	
Less than high school	56,560 (18.2)	1,539 (24.2)	<0.001
Household income, *n* (%)			
More than ₤52,000	68,134 (22.0)	812 (12.8)	
₤18,000–51,999	162,937 (52.5)	3,233 (50.9)	
Less than ₤18,000	79,237 (25.5)	2,310 (36.3)	<0.001
Employed, *n* (%)	290,720 (93.7)	6,043 (95.1)	<0.001
BMI, kg/m^2^	27.5 (4.7)	27.9 (4.8)	<0.001
SBP, mm Hg	142.2 (19.6)	144.6 (19.8)	<0.001
DBP, mm Hg	82.7 (10.6)	81.8 (10.7)	<0.001
Smoker, *n* (%)	147,383 (47.5)	3,287 (51.7)	<0.001
Heavy drinker, *n* (%)	104,850 (33.8)	2,137 (33.6)	0.797
Regular physical activity, *n* (%)	87,624 (28.2)	1,679 (26.4)	0.002
Healthy diet, *n* (%)	76,832 (24.8)	1,714 (27.0)	<0.001
Hypertension, *n* (%)	86,938 (28.0)	2,183 (34.4)	<0.001
Diabetes, *n* (%)	17,895 (5.8)	635 (10.0)	<0.001
Cancer, *n* (%)	27,328 (8.8)	660 (10.4)	<0.001

SES, socioeconomic status; BMI, body mass index; AMD, age-related macular degeneration; SBP, systolic pressure; DBP, diastolic pressure.

Table 2 summarized the baseline characteristics of study variables by SES subgroup. A total of 51,639 (16.3%), 163,441 (51.6%), and 101,583 (32.1%) were identified as High-SES, Medium-SES, and Low-SES group via the LCA model, respectively. Compared to participants with a high SES, individuals with a low SES were older and less often male. Moreover, they were less often of white ethnicity and had a higher BMI. Their lifestyle was also different: their diet was worse, they smoked more often, they drank less, and they were less regularly physically active. Lastly, they were more often diagnosed with hypertension, diabetes, and cancer.

**Table 2.** Characteristics at baseline and follow-up by socioeconomic status. Study variables were described using mean (SD) or *n* (%), appropriately.

Variable	High-SES	Medium-SES	Low-SES	Total participants	*P* value
*N*	51,639	16,3441	101,583	316,663	
Baseline					
Age, years	57.0 (4.9)	59.5 (5.4)	61.7 (5.2)	59.8 (5.5)	<0.001
Males, *n* (%)	27,877 (54.0)	79,624 (48.7)	44,882 (44.2)	152,383 (48.1)	<0.001
White ethnicity or race, *n* (%)	49,906 (96.6)	157,801 (96.5)	96,928 (95.4)	304,635 (96.2)	<0.001
Education, *n* (%)					
College or above	41,300 (80.0)	65,234 (39.9)	16,976 (16.7)	123,510 (39.0)	
High school or equivalent	10,339 (20.0)	89,299 (54.6)	35,416 (34.9)	135,054 (42.6)	
Less than high school	0 (0.0)	8,908 (5.5)	49,191 (48.4)	58,099 (18.3)	<0.001
Household income, *n* (%)					
More than ₤52,000	50,938 (98.6)	17,442 (10.7)	566 (0.6)	68,946 (21.8)	
₤18,000–51,999	701 (1.4)	145,999 (89.3)	19,470 (19.2)	166,170 (52.5)	
Less than ₤18,000	0 (0.0)	0 (0.0)	81,547 (80.3)	81,547 (25.8)	<0.001
Employed, *n* (%)	49,249 (95.4)	161,491 (98.8)	86,023 (84.7)	296,763 (93.7)	<0.001
BMI, kg/m^2^	26.7 (4.2)	27.4 (4.6)	28.2 (5.1)	27.5 (4.7)	<0.001
SBP, mm Hg	138.7 (18.7)	142.1 (19.4)	144.1 (20.1)	142.2 (19.6)	<0.001
DBP, mm Hg	82.5 (10.6)	82.8 (10.6)	82.5 (10.7)	82.7 (10.6)	<0.001
Smoker, *n* (%)	20,872 (40.4)	75,644 (46.3)	54,154 (53.3)	150,670 (47.6)	<0.001
Heavy drinker, *n* (%)	22,323 (43.2)	57,863 (35.4)	26,801 (26.4)	106,987 (33.8)	<0.001
Regular physical activity, *n* (%)	13,259 (25.7)	47,777 (29.2)	28,267 (27.8)	89,303 (28.2)	<0.001
Healthy diet, *n* (%)	13,517 (26.2)	41,314 (25.3)	23,715 (23.3)	78,546 (24.8)	<0.001
Hypertension, *n* (%)	11,258 (21.8)	44,383 (27.2)	33,480 (33.0)	89,121 (28.1)	<0.001
Diabetes, *n* (%)	1,754 (3.4)	8,140 (5.0)	8,636 (8.5)	18,530 (5.9)	<0.001
Cancer, *n* (%)	3,818 (7.4)	13,954 (8.5)	10,216 (10.1)	27,988 (8.8)	<0.001
Follow-up					
Incident AMD, *n* (%)	583 (1.13)	3,006 (1.84)	2,766 (2.72)	6,355 (2.01)	<0.001
Follow-up duration, years	12.5 (1.7)	12.3 (1.9)	12.0 (2.4)	12.3 (2.1)	<0.001

SES, socioeconomic status; BMI, body mass index; AMD, age-related macular degeneration; SBP, systolic pressure; DBP, diastolic pressure.

### Association analyses of SES inequity with incident AMD

Cox proportional hazards models were used to estimate the risk impact of SES inequity on incident AMD (Table 3). In the crude model, participants with a medium SES (HR [95% CI]: 1.66 [1.51 to 1.81]) and low SES (HR [95% CI]: 2.54 [2.33 to 2.78]) had a higher risk of incident AMD, compared to participants with a high SES. After adjusting for baseline age, sex, race, assessment center, BMI, histories of hypertension, diabetes, cancer, and healthy lifestyle score, this relationship was still significant. Medium-SES and Low-SES groups were both associated with a 10% (HR: 1.10, 95% CI: 1.01 to 1.21, *P* = 0.038) and 22% (HR: 1.22, 95% CI: 1.11 to 1.34, *P* < 0.001) increased risk for AMD, respectively. In addtion, these results remained consistent in males and females (Table 3).

**Table 3.** Hazard ratios (95% CIs) of socioeconomic status inequity with AMD

	Model 1 ^a^	Model 2 ^b^	Model 3 ^c^
Total		
High-SES	Reference	Reference	Reference
Medium-SES	1.66 (1.51, 1.81)	1.10 (1.01, 1.21)	1.10 (1.01, 1.21)
Low-SES	2.54 (2.33, 2.78)	1.21 (1.10, 1.34)	1.22 (1.11, 1.34)
Males		
High-SES	Reference	Reference	Reference
Medium-SES	1.56 (1.38, 1.77)	1.08 (0.95, 1.23)	1.08 (0.95, 1.23)
Low-SES	2.28 (2.00, 2.59)	1.18 (1.03, 1.36)	1.19 (1.03, 1.36)
Females		
High-SES	Reference	Reference	Reference
Medium-SES	1.72 (1.52, 1.95)	1.12 (0.98, 1.28)	1.12 (0.99, 1.28)
Low-SES	2.68 (2.37, 3.04)	1.25 (1.09, 1.43)	1.26 (1.10, 1.44)

^a^ Unadjusted for any covariates.

^b^ Adjusted for baseline age and gender (only for total), race, assessment center, BMI, self-report hypertension, self-report diabetes, and self-report cancer.

^c^ Adjusted for baseline age and gender (only for total), race, assessment center, BMI, self-report hypertension, self-report diabetes, self-report cancer, and healthy lifestyle scores.

SES, socioeconomic status; AMD, age-related macular degeneration; CI, confidence interval; BMI, body mass index.

Besides, we also evaluated the associations of SES factors with incident AMD (Table S2). After adjusting for the covariates mentioned above, both the lowest education level and the lowest household income were related with increased risk of incident AMD, with HRs and 95% CIs of 1.14 (1.06, 1.22) and 1.20 (1.11, 1.32), respectively. The results of stratified analyses were consistent with our main analysis (Table S3). Furthermore, a series of sensitivity analyses were conducted, and SES–AMD association was robust in all scenarios (Table S4).

### The impacts of healthy lifestyle in SES–AMD association

Subsequently, we explored the modification, mediation, and joint effects of healthy lifestyle score on the association between SES inequity and AMD, respectively.

**Fig. 1.**
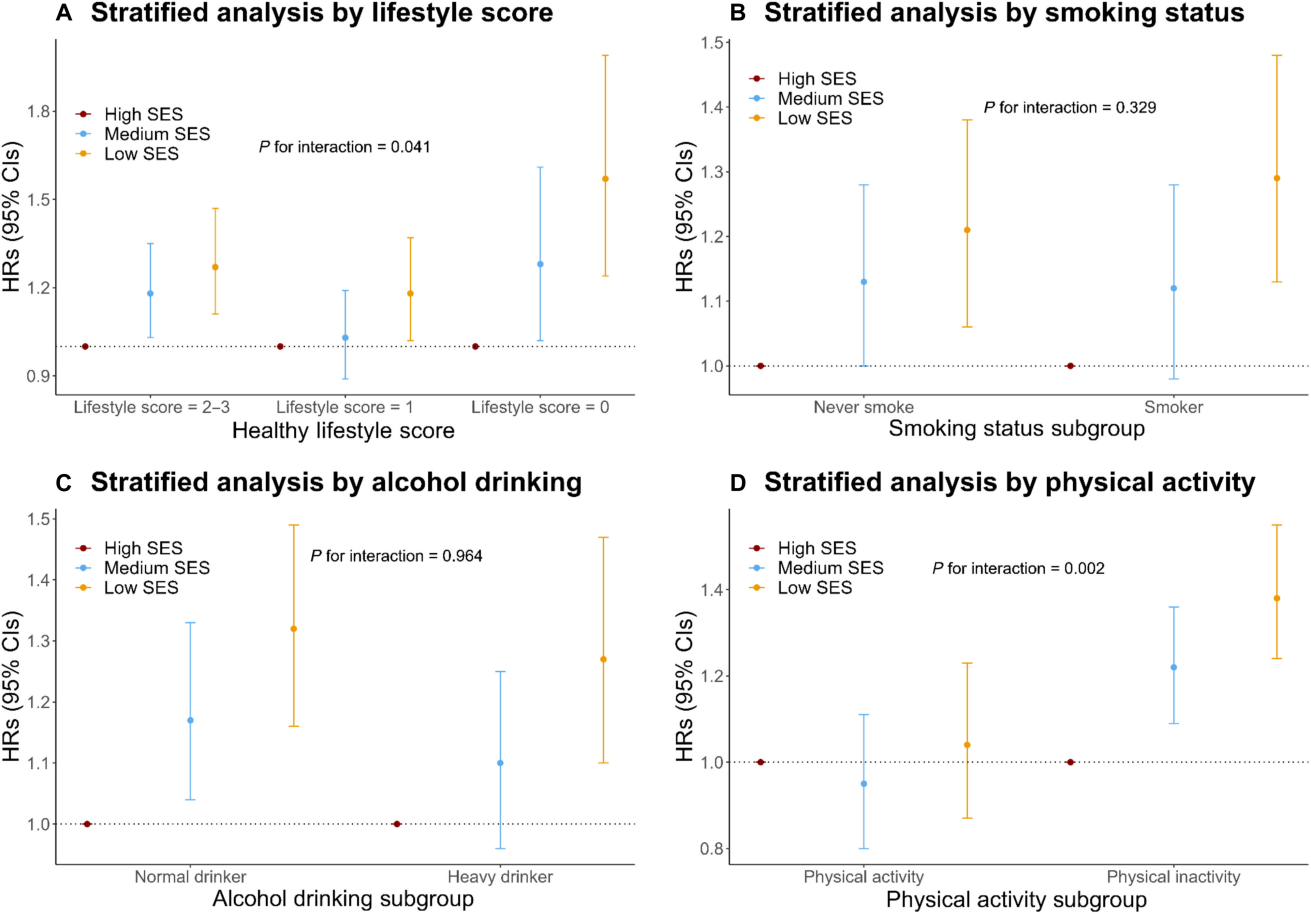
Associations of socioeconomic status and incident AMD by healthy lifestyle score and each behavior. The association between incident AMD and socioeconomic status stratified on healthy lifestyle score (A), smoking status (B), alcohol drinking (C), and physical activity (D). Circles represent hazard ratios; horizontal lines indicate corresponding 95% confidence intervals around hazard ratios. Hazard ratios were calculated using Cox proportional hazards models after adjusting for baseline age and gender, race, assessment center, BMI, self-report hypertension, self-report diabetes, and self-report cancer. Multiplicative interaction was evaluated using hazard ratios for the product term between SES (high vs low) and one-point decrease for the healthy lifestyle score, or risk behavior for each lifestyle behavior. AMD, age-related macular degeneration; SES, socioeconomic status; HR, hazard ratios; CI, confidence intervals; BMI, body mass index.

The incidence of AMD was increased with decreased SES, while an unhealthy lifestyle score would enhance this relationship (Figs. S2 and S3). The effect of SES inequity on AMD was significantly modified by healthy lifestyle score (*P* for interaction = 0.041), especially by PA (*P* for interaction = 0.002). Stratified analyses found that the impacts of medium SES and low SES on incident AMD varied widely across healthy lifestyle score categories, with HRs (95% CIs) of 1.18 (1.03, 1.35) and 1.27 (1.11, 1.47) for those with 2 to 3 points and 1.28 (1.02, 1.61) and 1.57 (1.24, 1.99) for those with 0 points, respectively (Fig. 1). Furthermore, SES–AMD association was unobserved in those with PA (*P* > 0.05) but was significant in physical inactivity group (*P* < 0.001 for all), with HRs of 1.22 and 1.38 for the Medium-SES and Low-SES group, respectively (Fig. 1). Similar results were found in the household income analyses, but these modification effects were not significant on the education–AMD association (Figs. S4 and S5).

Table 4 presented the results of mediation analyses. After adjusting for baseline age, sex, race, assessment center, BMI, and history of diseases, the mediation effect of healthy lifestyle score between SES inequity and incident AMD was observed, and particularly smoking and alcohol drinking were important mediators of this association. Smoking mediated 11.3% (95% CI: 6.56 to 23.0, *P* < 0.001) of the association between a low SES and AMD. The counterpart effect of alcohol drinking was −9.59% (−23.2, −4.00), which was defined as suppression effect, indicating that alcohol consumption would reduce 9.59% (*P* < 0.001) of the protective effect of high SES on incident AMD. Table S5 showed that the analyses about SES factors were in line with the main results, except for the positive mediation effect of healthy lifestyle score on education–AMD association (3.28%, 95% CI: 1.01 to 6.00).

**Table 4.** Mediation effects of healthy lifestyle behaviors on the SES–AMD associations ^a^. All models were adjusted for baseline age and gender, race, assessment center, BMI, self-report hypertension, self-report diabetes, and self-report cancer. The odds ratios and 95% confidence intervals were the measure of direct effect and total effect. Prop = the proportion of the total effect explained by the mediator. A proportion of mediation and 95% CI that did not contain zero indicated significant mediation effect.

Mediators	Direct effect	*P* value	Total effect	*P* value	Prop mediation (%)	*P* value
Healthy lifestyle score	1.19 (1.08, 1.31)	<0.001	1.18 (1.07, 1.30)	<0.001	−3.93 (−9.31, −2.00)	<0.001
Smoking	1.17 (1.06, 1.28)	<0.001	1.18 (1.07, 1.30)	<0.001	11.3 (6.56, 23.0)	<0.001
Alcohol drinking	1.20 (1.09, 1.32)	<0.001	1.18 (1.07, 1.30)	<0.001	−9.59 (−23.2, −4.00)	<0.001
Physical inactivity	1.18 (1.07, 1.30)	<0.001	1.18 (1.07, 1.30)	<0.001	−1.25 (−4.83, 1.00)	0.199

SES, socioeconomic status; AMD, age-related macular degeneration; CI, confidence interval.

^a^ Only the results comparing the low with high socioeconomic status were reported.

**Fig. 2.**
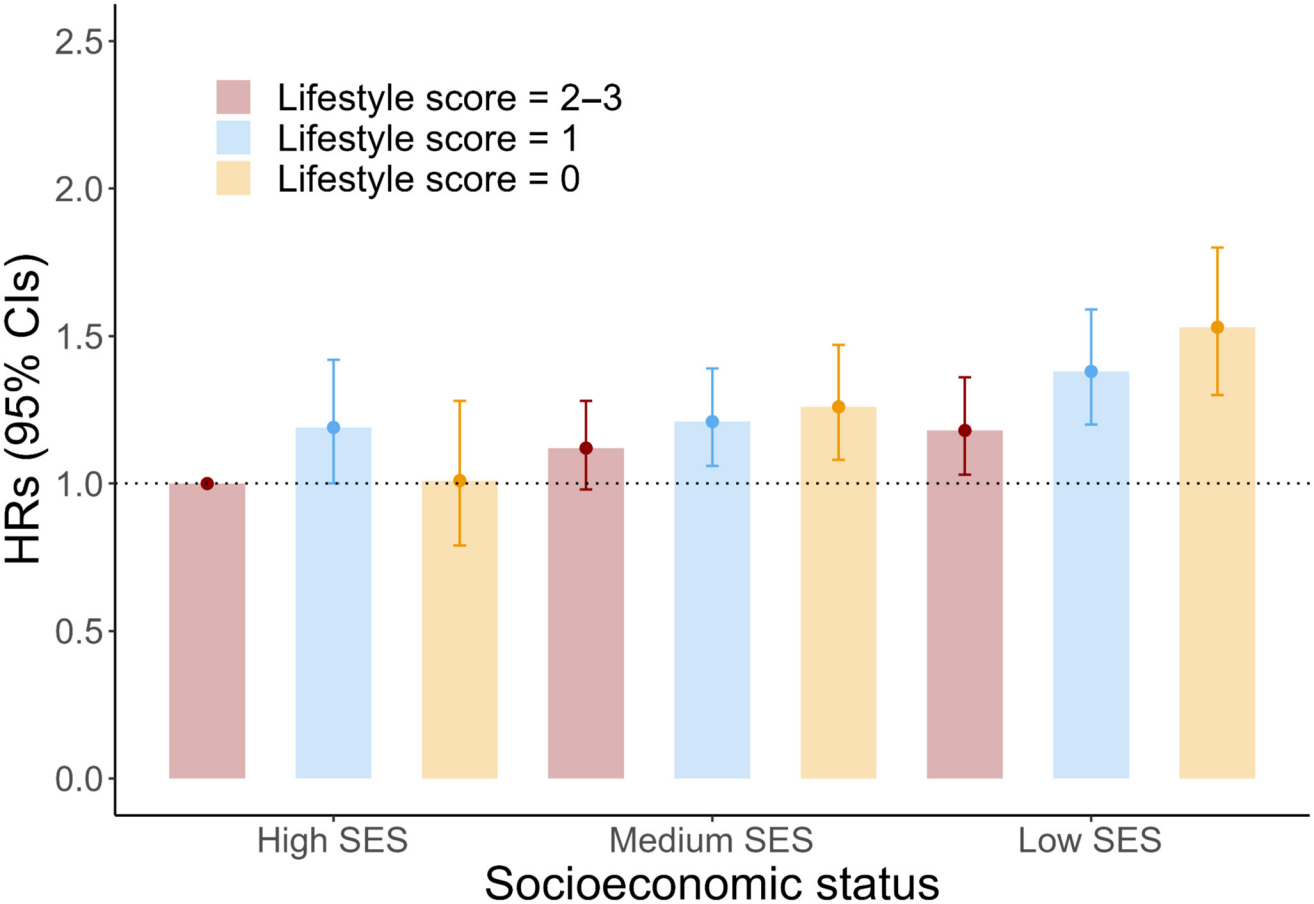
Joint associations of healthy lifestyle score and socioeconomic status with incident AMD. Circles represent hazard ratios; horizontal lines indicate corresponding 95% confidence intervals around hazard ratios. Hazard ratios were calculated using Cox proportional hazards models after adjusting for baseline age and gender, race, assessment center, BMI, self-report hypertension, self-report diabetes, and self-report cancer. Those in the High-SES group with a lifestyle score of 2 to 3 were treated as the reference. AMD, age-related macular degeneration; SES, socioeconomic status; HR, hazard ratios; CI, confidence intervals; BMI, body mass index.

Figure 2 showed the joint association of SES inequity and healthy lifestyle score on incident AMD. After adjusting for covariables in model 2, the HR (95% CI) for participants in the Low-SES group with 0 points of healthy lifestyle score was 1.53 (1.30, 1.80), compared with those in the High-SES group and 2 to 3 points of healthy lifestyle score (*P* for trend <0.001). Results were not substantially changed in education level and household income subgroup analyses, with the HRs and 95% CIs of 1.36 (1.18, 1.57) for education analysis and 1.55 (1.32, 1.81) for income analysis, respectively (Fig. S6). Finally, the main results of the current study were summarized in Table S6.

## Discussion

Using a population-based data from the UK Biobank, the current study identified a significant association between SES inequality and incident AMD, and this relationship was modified by lifestyles, especially by PA. Notably, cigarette smoking would mediate the low SES–AMD association, while the protective effect of high SES was suppressed due to their higher consumption of alcohol. Our results underscore the universal benefits of positive lifestyle adjustments. Specifically, promoting increased PA and smoking cessation is crucial for individuals with low SES, while reducing alcohol consumption should be prioritized among those with high SES, to mitigate their susceptibility to AMD.

Our results align with previous studies, proving that SES inequity correlates with an elevated risk of developing AMD later in life [11–18]. Researchers have estimated the impact of low education and household income on AMD, and the odds ratios ranged from 1.32 to 4.44 [11–16]. However, their focus only limited to education or income singly, ignoring the interrelations of socioeconomic factors [11–16]. Additionally, the temporal link between SES and AMD remains inadequately established in these studies [11–16], primarily due to their cross-sectional or case-control designs. In contrast, other studies have reported no discernable connection between SES inequity and AMD [9,10]. These divergent findings might stem from a limited sample size, and insufficient follow-up periods for AMD assessment. It is pivotal to address the latter point because AMD often manifests a lengthy prodromal phase before diagnosis, and studies with short follow-up periods are particularly susceptible to reverse causation. Our prospective cohort study ameliorated limitations above. We enrolled a sizable cohort with 3,877,557 person-years of follow-up, containing 6,355 AMD cases, and a comprehensive SES evaluation was conducted via LCA models. Consequently, our results provide compelling evidence that low SES is a significant risk factor for AMD, underscoring the hazard of SES inequity for the ocular health. Besides, we also admit that UK Biobank is a relatively young cohort for AMD [27–29]. Further studies based on elder population is needed to verify the findings of the current study.

We also observed the risk of SES inequity on incident AMD was significantly modified by healthy lifestyles, especially by PA. Previous evidence has well-established that physical inactivity independently increased the risk of AMD, amplifying the likelihood by 2 to 3 folds due to its negative effects on inflammation status, oxidizing stress, and other factors [30–32]. Additionally, Wang *et al*. [33] illustrated that low SES was significantly correlated with physical inactivity, with odds ratios ranging from 1.18 to 6.14. Hence, it seems reasonable that physical inactivity would enhance the risk impact of low SES on AMD. Researchers have illustrated that healthy lifestyle score could modify the low SES–diseases associations [21,23]. However, their studies revealed that the low SES group was significantly associated with an increased risk of health outcomes in each healthy lifestyle score group [21,23]. Failure to consider the specific impact of each behavior may lead to an underestimation of the modification effect, as some behaviors may not exhibit significant modification effects. Our results firstly report that a modifiable factor could counteract the hazard of SES inequity on AMD, which is an originally inalterable association. PA should be encouraged to reduce the risk of AMD, especially in those presenting with low SES.

Notably, healthy lifestyle score would mediate the pathway linking SES inequity to AMD, and the effects of smoking and alcohol drinking were inverse. Previous studies have affirmed that low-SES group smoked more frequently, compared with those in high-SES group (30.8% vs 26.5%) [34]. In addition, the risk of smoking on incident AMD has been estimated widely [35,36]. However, these studies usually explored the relationship of SES, smoking, and AMD separately, leaving the low SES-smoking-AMD pathway still ambiguous. Our mediation analysis indicated that smoking might bridge the gap between low SES and AMD, with an approximately 10% mediation proportion. In contrast, as the recognized risk factor for AMD [37,38], the effect of alcohol drinking was mainly on those with high SES. Our study found that the proportion of alcohol drinking was the greatest in high-SES group, and about 10% of the protective effect of high SES on AMD prevention was removed due to the alcohol consumption. A previous study using UK Biobank has clarified that healthy lifestyle score mediated 3.7% to 5.1% of the SES–mortality association [21]. However, the inverse effects of smoking and alcohol drinking were not reported, which would cause a miscalculation of the mediation proportion. Our results underscore cessation of smoking and alcohol drinking should be targeted emphasized in those with low and high SES, respectively, to reduce their susceptibility to AMD. Moreover, the heightened AMD risk could not be solely attributable to lifestyle behaviors. It is crucial to perform more research to delve deeper into the potential link between SES inequity and AMD.

Our study presents several major strengths. Firstly, this prospective cohort recruited a large sample size with adequate follow-up duration, which ensured us to perform complex stratified and joint analyses with sufficient statistical power as well as to observe adequate participants developing AMD. Secondly, using LCA allowed us to construct an overall SES variable to systematically estimate the relationship between SES inequity and AMD. Thirdly, the roles of the healthy lifestyle score and each behavior in SES–AMD association were evaluated, providing evidence for the formulation and implementation of public health strategies aimed at reducing the risk of AMD.

However, several limitations warrant consideration. Firstly, the current study only used the baseline characteristics to define SES inequity, ignoring the changing patterns during adulthood. The accumulated life-course effect of SES on AMD could not be evaluated. Besides, the information on socioeconomic and lifestyle variables was collected at the same survey, and the temporal sequence was unclear. Although previous study has reported that SES was longitudinally associated with lifestyle behaviors [33], the mediation effects should be cautiously interpreted. Secondly, the UK Biobank cohort is primarily composed of middle-aged participants with a predominantly Caucasian ethnic background. The results of subgroup analyses, especially among the non-Whites (*N* = 11,200), may lack sufficient statistical power. Furthermore, AMD is an age-related disease, so we excluded those below 50 years old. Thus, findings from our analyses may not be generalizable to other age groups [21]. Thirdly, those with missing data were excluded in our study, introducing potential selection bias. However, we performed a sensitivity analysis using a multiple imputation method. The results were in consistent with our main findings. Fourthly, the alcohol drinking was considered in whole as a risky behavior, ignoring the effects of different kinds of alcohol in different diet patterns, which also limited our results to generalize. Fifthly, the current study used logistic regression to explore the mediation effect of lifestyle on SES–AMD association, which was not one-on-one comparable with the Cox proportional hazards models. Sixthly, the data about subtype of AMD was missing in our study, which restricted us to explore the specific impact of SES inequity on dry AMD or wet AMD. Finally, although we have adjusted as many covariates as we can in our regression models, findings of our study might be influenced by the unobserved confounders, such as genetic and environmental factors.

In conclusion, our study demonstrated a significant association between SES inequity and the risk of AMD, and this association could be diminished by PA. PA should be encouraged to limit the effect of SES inequity on incident AMD. Reduction in smoking and alcohol drinking should be advocated in those with either low or high SES to lower their vulnerability to AMD.

## Ethical Approval

All participants in the UK Biobank provided the signed consent (www.ukbiobank.ac.uk), and UK Biobank study was approved by the North West Multi-Centre Research Ethics Committee (Ref: 11/NW/0382).

## Data Availability

UK Biobank data are available to all researchers for health-related research and public interest (www.ukbiobank.ac.uk).

## Supplementary Materials

Supplementary 1 Sections S1 and S2 Tables S1 to S6 Figs. S1 to S6
