# Supplementary material for: Associations of Socioeconomic Status Inequity with Incident Age-related Macular Degeneration in Middle-Aged and Elderly Population
Source: Health Data Sci. 2024 Jul 1;4:0148. doi: 10.34133/hds.0148 (PMC11214909; doi:10.34133/hds.0148)
Supplement: Supplementary 1 — Sections S1 and S2 Tables S1 to S6 Figs. S1 to S6 [file hds.0148.f1.docx]

**Associations of** **Socioeconomic Status Inequity with Incident** **Age-related Macular Degeneration in Middle-aged and Elderly Population**

**Supplementary file 1.** Assessment of socioeconomic status using latent class analysis

**Supplementary file 2.** Assessment of healthy lifestyle score and each behavior

**Table S1.** Baseline characteristics of participants included and excluded

**Table S2.** Associations of each individual socioeconomic factor with incident AMD

**Table S3.** Associations of socioeconomic status with incident AMD: subgroup analyses

**Table S4.** Sensitivity analyses for the associations between socioeconomic status and AMD

**Table S5.** Mediation effect of healthy lifestyle on the associations of SES factors and incident AMD: subgroup analyses

**Table S6.** The summary of main results

**Figure S1.** Flowchart of the included participants

**Figure S2.** Incidence of AMD across different combined categories of SES and lifestyle score

**Figure S3.** Incidence of AMD across different combined categories of SES and each lifestyle factor

**Figure S4.** Associations of education level and household income and incident AMD by different healthy lifestyle score

**Figure S5.** Associations of socioeconomic status factors and incident AMD by different lifestyle behavior subgroup

**Figure S6.** Joint associations of healthy lifestyle score and socioeconomic factors with incident AMD

**Supplementary file 1**

**Assessment of socioeconomic status using latent class analysis**

Education qualification, total household income before tax, and employment status were included to estimate an overall SES sub-group in the UK Biobank. Healthy insurance was not considered in the current study due to the National Health Service. In the process of latent class analysis (LCA), education level and household income were not regrouped because of the large sample size in the UK Biobank and the failure of model convergence, which was in line with previous studies^1,2^. Seven levels according to education qualification were recorded, including “College or University degree”, “A levels/AS levels or equivalent”, “O levels/GCSEs or equivalent”, “CSEs or equivalent”, “NVQ or HND or HNC or equivalent”, “Other professional qualifications”, and “None of the above” (equivalent to or less than high school diploma). Individuals were divided into five sub-groups due to the household income, containing “less than ₤18,000”, “₤18,000 to 30,999”, “₤31,000 to 51,999”, “₤52,000 to 100,000”, and “greater than ₤100,000”. In the UK Biobank, information on specific occupation were not collected at baseline, so that we regroup individuals into two sub-groups based on their employment status, involving employed (those in paid employment or self-employed, retired, doing unpaid or voluntary work, or being full or part-time students) and unemployed.

LCA model was used to identify the SES subgroup via the three variables above. LCA is an unsupervised clustering method, which uses multiple observed categorical variables to generate an unmeasured variable (i.e., latent variable) with a set of mutually exclusive latent classes ^[1]^. We fitted different LCA models with 2-10 latent classes. The best-fitting model for SES patterns was selected according to the Akaike information criterion (AIC), Bayesian information criterion (BIC), and likelihood ratio statistic (G^2^) while ensuring that each latent class has an acceptable posterior probability (more than 0.7). LCA models were constructed by “*poLCA*” package in R (version 4.1.3).

In the current study, models failed to converge when the class number is greater than three. In the three-latent class model, the *G*^2^ statistic was 2341, AIC was 2843859, and BIC was 2844242. Average posterior probabilities, the prevalence of latent classes, and item-response probabilities in the three-latent class model were shown below.

**Table 1 for supplementary method.** Average posterior probabilities, prevalence of latent classes, and item-response probabilities in three-latent class model in the UK Biobank

| Item | Latent class 1 | Latent class 2 | Latent class 3 |
| --- | --- | --- | --- |
| APP | 0.77 | 0.93 | 0.80 |
| Prevalence* | 0.52 | 0.28 | 0.20 |
| More than ₤100,000 | 0.00 | 0.01 | 0.23 |
| ₤52,000~100,000 | 0.17 | 0.01 | **0.54** |
| ₤31,000~51,999 | **0.42** | 0.04 | 0.24 |
| ₤18,000~30,999 | 0.41 | 0.22 | 0.00 |
| Less than ₤18,000 | 0.00 | **0.72** | 0.00 |
| College or university degree | **0.30** | 0.15 | **0.72** |
| A/AS levels or equivalent | 0.13 | 0.08 | 0.13 |
| O/GCSEs level or equivalent | 0.27 | 0.21 | 0.09 |
| CSEs or equivalent | 0.07 | 0.06 | 0.01 |
| NVQ/HND/HNC or equivalent | 0.08 | 0.08 | 0.02 |
| Other professional qualifications | 0.06 | 0.05 | 0.03 |
| None of the above | 0.08 | **0.36** | 0.00 |
| Employed | **0.98** | **0.84** | **0.96** |
| Unemployed | 0.02 | 0.16 | 0.04 |

APP, average posterior probabilities.

* Prevalence indicated the prevalence of each latent class.

As shown above, individuals in latent class 1 were more likely college or university degree, with ₤31,000~51,999 of total household income before tax, and employed, which could be considered as “Medium-SES”. Those in latent class 2 presented relatively high proportion of less than high school (i.e., none of the above), less than ₤18,000 of household income and unemployment (16% vs 2% and 4%), and was defined as “Low-SES”. Furthermore, ₤52,000~100,000 of household income, college or university degree, and employment status were prevalent in latent class 3, which could be defined as “High-SES”. Finally, three-latent classes were identified, which respectively represented a high, medium, and low SES according to the item-response probabilities.

Notably, we further regrouped education level and household income in the variable description and SES factors sub-group analyses. In term of education qualification, individuals were divided into college or above (i.e., college or university degree and other professional qualifications), high school or equivalent (including A/AS levels or equivalent, O/GCSEs level or equivalent, CSEs or equivalent, and NVQ/HND/HNC or equivalent), and less than high school (i.e., none of the above). For total household income before tax, three categories were regrouped, including high income (more than ₤52,000), medium income (₤18,000~51,999), and low income (less than ₤18,000).

**Supplementary file 2**

**Assessment of healthy lifestyle score and each behavior**

**Cigarette smoking**: Participants were asked about their smoking status, including never smoking, previous smoking, and current smoking. In the current study, cigarette smoking was defined as previous or current smoking, and was considered as risk behavior.

**Alcohol drinking**: In the UK Biobank, participants were asked about the frequency of drinking alcohol, containing (almost) daily, three or four times a week, once or twice a week, one to three times a month, special occasions only, never, and prefer not to answer. Those who reported to drink alcohol would be asked about how much red wine (glasses), white wine (glasses), beer or cider (pints), spirits or liqueurs (standard measures), fortified wine (glasses), and other alcoholic drinks (glasses) they consumed in an average month or week. We used the information to calculate the average units of alcohol each participant drank daily. A healthy level was defined as daily consumption of one drink or fewer for women and two drinks or fewer for men, according to the dietary guidelines in the UK (8g alcohol/ethanol).

**Regular physical activity**: Frequency and time for physical activity (PA) were asked for individuals in the UK Biobank. In the current study, regular PA was defined to meet one of the following: (i) from the perspective of frequency, to engage in vigorous physical activity for at least one day and moderate activity for at least five days per week; (ii) from the perspective of time, to exercise of vigorous activity for at least 75 min or moderate activity for 150 min per week.

**Healthy diet**: Six components were selected to defined diet pattern, including (i) adequate consumption of fruit, (ii) vegetables, (iii) fish, but (iv) reduced consumption of processed and (v) unprocessed meats, and (vi) whole grains. The specific definition for each pattern was shown below, and following at least four factors was defined as healthy diet.

**Table 2 for supplementary method.** Components of more recent dietary recommendations for health.

| **Diet component** | **Intake goal** | **Definition of amount of per serving** |
| --- | --- | --- |
| Fruits | ≥4 servings/day | 1. fresh fruit: 1 piece;  2. dried fruit: 5 pieces;  3. cooked/raw vegetables: 3 heaped tablespoons;  4. bran/oat/muesli cereal: 1 bowl/day:  5.wholemeal/wholegrain bread: 1 slice/day |
| Vegetables | ≥4 servings/day |  |
| Fish | ≥2 times/week |  |
| Processed meat | ≤1 time/week |  |
| Unprocessed red meat | ≤1.5 times/week |  |
| Whole grain | ≤3 servings/day |  |

**Selection of lifestyle factors**

For each factor, a healthy level was assigned 1 point while 0 points for the unhealthy level. Furtherly, Cox proportional hazard regression models were used to explore the relationship between these four factors and incident age-related macular degeneration (AMD), and the results were shown below.

**Figure for supplementary method.** The relationship between four healthy lifestyle factors and incident AMD*


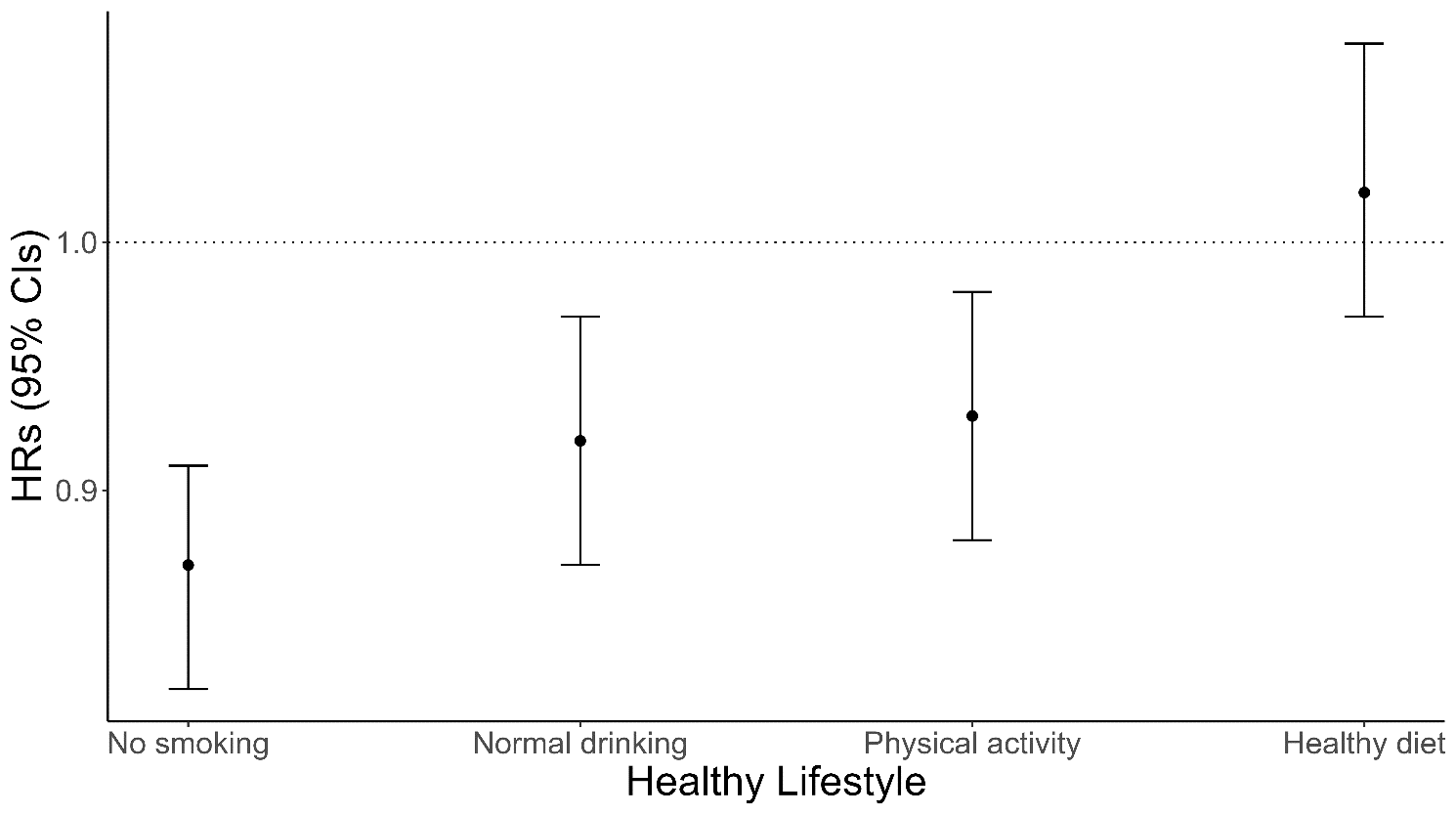


* All models were adjusted for baseline age and gender, race, assessment center, BMI, self-report hypertension, self-report diabetes, self-report cancer, and SES subgroup.

As shown above, never smoking, normal alcohol drinking and physical activity were associated with a decreased risk of incident AMD. In the current setting, diet was not associated with incident AMD. Thus, we established a total score of healthy lifestyle behavior based on these three significant risk factors. However, in the sensitivity analyses, we redefined the healthy lifestyle score through including healthy diet to examine the robustness of observations.

**Reference**

1. Zhang YB, Chen C, Pan XF, Guo J, Li Y, Franco OH, Liu G, Pan A. Associations of healthy lifestyle and socioeconomic status with mortality and incident cardiovascular disease: two prospective cohort studies. BMJ. 2021 Apr 14;373:n604.
2. Ye X, Wang Y, Zou Y, Tu J, Tang W, Yu R, Yang S, Huang P. Associations of socioeconomic status with infectious diseases mediated by lifestyle, environmental pollution and chronic comorbidities: a comprehensive evaluation based on UK Biobank. Infect Dis Poverty. 2023 Jan 30;12(1):5.

**Table S1.** Baseline characteristics of participants included and excluded

| Variables | Included | Excluded | *P* value |
| --- | --- | --- | --- |
| N | 316663 | 185713 |  |
| Age, years | 59.8 (5.5) | 50.9 (8.7) | <0.001 |
| Males, n (%) | 152383 (48.1) | 76688 (41.3) | <0.001 |
| White ethnicity or race, n (%) | 304635 (96.2) | 167945 (90.4) | <0.001 |
| **Education, n (%)** |  |  |  |
| College or above | 123510 (39.0) | 63398 (34.1) |  |
| High school or equivalent | 135054 (42.6) | 85024 (45.8) |  |
| Less than high school | 58099 (18.3) | 27159 (14.6) | <0.001 |
| **Household income, n (%)** |  |  |  |
| More than ₤52,000 | 68946 (21.8) | 40221 (21.7) |  |
| ₤18,000~51,999 | 166170 (52.5) | 52721 (28.4) |  |
| Less than ₤18,000 | 81547 (25.8) | 15630 (8.4) | <0.001 |
| Employed, n (%) | 296763 (93.7) | 160923 (86.7) | <0.001 |
| BMI, kg/m^2^ | 27.5 (4.7) | 27.3 (5.0) | <0.001 |
| SBP, mm Hg | 142.2 (19.6) | 135.5 (19.1) | <0.001 |
| DBP, mm Hg | 82.7 (10.6) | 81.4 (10.8) | <0.001 |
| Smoker, n (%) | 150670 (47.6) | 75303 (40.5) | <0.001 |
| Alcohol drinker, n (%) | 106987 (33.8) | 55118 (29.7) | <0.001 |
| Regular physical activity, n (%) | 89303 (28.2) | 51491 (27.7) | <0.001 |
| Healthy diet, n (%) | 78546 (24.8) | 39496 (21.3) | <0.001 |
| Hypertension, n (%) | 89121 (28.1) | 32058 (17.3) | <0.001 |
| Diabetes, n (%) | 18530 (5.9) | 7865 (4.2) | <0.001 |
| Cancer, n (%) | 27988 (8.8) | 10622 (5.7) | <0.001 |

Variables were described using mean (SD), and n (%), as appropriate.

**Abbreviations:** BMI, body mass index; SBP, systolic pressure; DBP, diastolic pressure.

**Table S2.** Associations of each individual socioeconomic factor with incident AMD

|  | Model 1^*^ | Model 2^**^ | Model 3^***^ |
| --- | --- | --- | --- |
| **Education attainment** | | | |
| College or above | Reference | Reference | Reference |
| High school or equivalent | 1.09 (1.03, 1.16) | 1.03 (0.97, 1.09) | 1.03 (0.97, 1.09) |
| Less than high school | 1.53 (1.43, 1.63) | 1.14 (1.07, 1.22) | 1.14 (1.06, 1.22) |
| **Household income** | | | |
| More than ₤52,000 | Reference | Reference | Reference |
| ₤18,000~51,999 | 1.68 (1.56, 1.82) | 1.08 (0.99, 1.17) | 1.09 (1.00, 1.18) |
| Less than ₤18,000 | 2.55 (2.35, 2.76) | 1.19 (1.09, 1.30) | 1.20 (1.11, 1.32) |

^*^ Unadjusted for any covariates.

^**^ Adjusted for baseline age and gender, race, assessment center, BMI, self-report hypertension, self-report diabetes, and self-report cancer.

^***^ Adjusted for baseline age and gender, race, assessment center, BMI, self-report hypertension, self-report diabetes, self-report cancer, and healthy lifestyle scores.

**Abbreviations:** AMD, age-related macular degeneration; BMI, body mass index.

**Table S3.** Associations of socioeconomic status with incident AMD: subgroup analyses

|  | Model 1* | Model 2** | Model 3*** |
| --- | --- | --- | --- |
| **Whites** (N = 304635) | | | |
| High-SES | Reference | Reference | Reference |
| Medium-SES | 1.65 (1.51, 1.81) | 1.08 (0.99, 1.19) | 1.09 (0.99, 1.19) |
| Low-SES | 2.55 (2.32, 2.79) | 1.20 (1.08, 1.32) | 1.20 (1.09, 1.33) |
| **Non-whites** (N = 11200) | | | |
| High-SES | Reference | Reference | Reference |
| Medium-SES | 1.69 (1.05, 2.72) | 1.47 (0.90, 2.40) | 1.47 (0.90, 2.40) |
| Low-SES | 2.31 (1.44, 3.71) | 1.60 (0.98, 2.63) | 1.60 (0.97, 2.63) |
| **Normal weight** (N = 99155) | | | |
| High-SES | Reference | Reference | Reference |
| Medium-SES | 1.71 (1.48, 1.99) | 1.13 (0.97, 1.32) | 1.14 (0.97, 1.33) |
| Low-SES | 2.42 (2.07, 2.82) | 1.20 (1.02, 1.42) | 1.21 (1.02, 1.42) |
| **Overweight or obesity** (N = 215996) | | | |
| High-SES | Reference | Reference | Reference |
| Medium-SES | 1.62 (1.45, 1.81) | 1.09 (0.97, 1.22) | 1.09 (0.97, 1.22) |
| Low-SES | 2.56 (2.29, 2.86) | 1.23 (1.09, 1.38) | 1.24 (1.10, 1.39) |
| **60 years or older** (N = 152831) | | | |
| High-SES | Reference | Reference | Reference |
| Medium-SES | 1.23 (1.12, 1.43) | 1.21 (1.07, 1.37) | 1.21 (1.07, 1.38) |
| Low-SES | 1.61 (1.43, 1.82) | 1.45 (1.28, 1.65) | 1.47 (1.29, 1.66) |
| **Less than 60 years** (N = 163832) | | | |
| High-SES | Reference | Reference | Reference |
| Medium-SES | 1.28 (1.11, 1.46) | 1.18 (1.03, 1.35) | 1.18 (1.03, 1.35) |
| Low-SES | 1.70 (1.47, 1.97) | 1.42 (1.22, 1.65) | 1.41 (1.21, 1.65) |

^*^ Unadjusted for any covariates.

^**^ Adjusted for baseline age and gender, race, assessment center, BMI, self-report hypertension, self-report diabetes, and self-report cancer (not including the covariate that was used to stratify the population).

^***^ Adjusted for baseline age and gender, race, assessment center, BMI, self-report hypertension, self-report diabetes, self-report cancer, and healthy lifestyle scores (not including the covariate that was used to stratify the population).

Overweight/Obesity: BMI >= 25 kg/m^2^; Normal weight: BMI < 25 kg/m^2^.

**Abbreviations:** SES, socioeconomic status; AMD, age-related macular degeneration; BMI, body mass index.

**Table S4.** Sensitivity analyses for the associations between socioeconomic status and AMD

| **Analyses** | **High-SES** | **Medium-SES** | **Low-SES** | ***P* for trend** |
| --- | --- | --- | --- | --- |
| Weighted lifestyle score | Reference | 1.10 (1.01, 1.20) | 1.22 (1.11, 1.34) | <0.001 |
| Lifestyle score including BMI | Reference | 1.10 (1.01, 1.21) | 1.22 (1.11, 1.34) | <0.001 |
| Lifestyle score including diet | Reference | 1.10 (1.00, 1.21) | 1.22 (1.11, 1.34) | <0.001 |
| Excluding individuals with chronic diseases | Reference | 1.08 (0.96, 1.22) | 1.19 (1.05, 1.35) | 0.002 |
| Excluding AMD occurred within the first two years | Reference | 1.11 (1.01, 1.22) | 1.23 (1.11, 1.35) | <0.001 |
| Adjusted for TDI additionally | Reference | 1.10 (1.00, 1.21) | 1.20 (1.09, 1.32) | <0.001 |
| Multiple imputation | Reference | 1.11 (1.04, 1.18) | 1.21 (1.12, 1.30) | <0.001 |
| Adjusted for blood pressure | Reference | 1.11 (1.01, 1.22) | 1.23 (1.11, 1.36) | <0.001 |
| Adjusted for total cholesterol | Reference | 1.10 (1.00, 1.21) | 1.22 (1.10, 1.34) | <0.001 |
| Competing risk analysis | Reference | 1.12 (1.02, 1.23) | 1.25 (1.13, 1.37) | <0.001 |

* All analyses were adjusted for baseline age and gender, race, assessment center, BMI, self-report hypertension, self-report diabetes, self-report cancer, and healthy lifestyle score.

**Abbreviations**: BMI, body mass index; SES, socioeconomic status; AMD, age-related macular degeneration; TDI, Townsend deprivation index.

**Table S5.** Mediation effect of healthy lifestyle on the associations of SES factors and incident AMD: subgroup analyses*.

| Mediators | Direct effect | *P* value | Total effect | *P* value | Prop Mediation (%) | *P* value |
| --- | --- | --- | --- | --- | --- | --- |
| **Education** | | | | | | |
| Healthy lifestyle score | 1.11 (1.04, 1.19) | 0.002 | 1.12 (1.04, 1.20) | 0.001 | 3.28 (1.01, 6.00) | <0.001 |
| Smoking | 1.10 (1.03, 1.18) | 0.006 | 1.12 (1.04, 1.20) | 0.001 | 11.8 (3.55, 26.0) | <0.001 |
| Alcohol drinking | 1.12 (1.05, 1.20) | 0.001 | 1.12 (1.04, 1.20) | 0.001 | -5.32 (-12.7, -1.00) | <0.001 |
| Physical inactivity | 1.12 (1.04, 1.19) | 0.002 | 1.12 (1.04, 1.20) | 0.001 | -0.31 (-1.32, 0.00) | 0.142 |
| **Household income** | | | | | | |
| Healthy lifestyle score | 1.17 (1.07, 1.28) | <0.001 | 1.16 (1.06, 1.27) | 0.001 | -7.27 (-19.2, -3.00) | <0.001 |
| Smoking | 1.15 (1.05, 1.25) | 0.002 | 1.16 (1.06, 1.27) | 0.001 | 12.5 (3.87, 44.0) | <0.001 |
| Alcohol drinking | 1.18 (1.08, 1.28) | <0.001 | 1.16 (1.06, 1.27) | 0.001 | -14.7 (-70.8, -5.00) | <0.001 |
| Physical inactivity | 1.16 (1.06, 1.27) | <0.001 | 1.16 (1.06, 1.27) | 0.001 | -0.57 (-4.40, 1.00) | 0.339 |

*Only the results comparing the low with high socioeconomic factor are reported.

All analyses were adjusted for baseline age and gender, race, assessment center, BMI, self-report hypertension, self-report diabetes, and self-report cancer.

The odds ratios and 95% confidence intervals were the measure of direct effect and total effect. Prop = the proportion of the total effect explained by the mediator. A proportion of mediation and 95% CI did not contain zero indicated significant mediation effect.

**Abbreviations:** SES, socioeconomic status; AMD, age-related macular degeneration; BMI, body mass index.

**Table S6**. The summary of main results.

|  | **Main results** |
| --- | --- |
| **Association** | Low SES was associated with increasing risk of incident AMD. |
|  | Both low education and low income was associated with increasing risk of incident AMD. |
| **Modification effects** | Lifestyle, especially PA, was modified the SES-AMD association. |
|  | Lifestyle, especially PA, was modified the income-AMD association. |
| **Mediation effects** | Smoking mediated the association between low SES or SES factors and AMD. |
|  | Alcohol drinking suppressed the protective effect of high SES or SES factors on AMD. |

SES factors include education level and household income.

**Abbreviations:** SES, socioeconomic status; AMD, age-related macular degeneration.


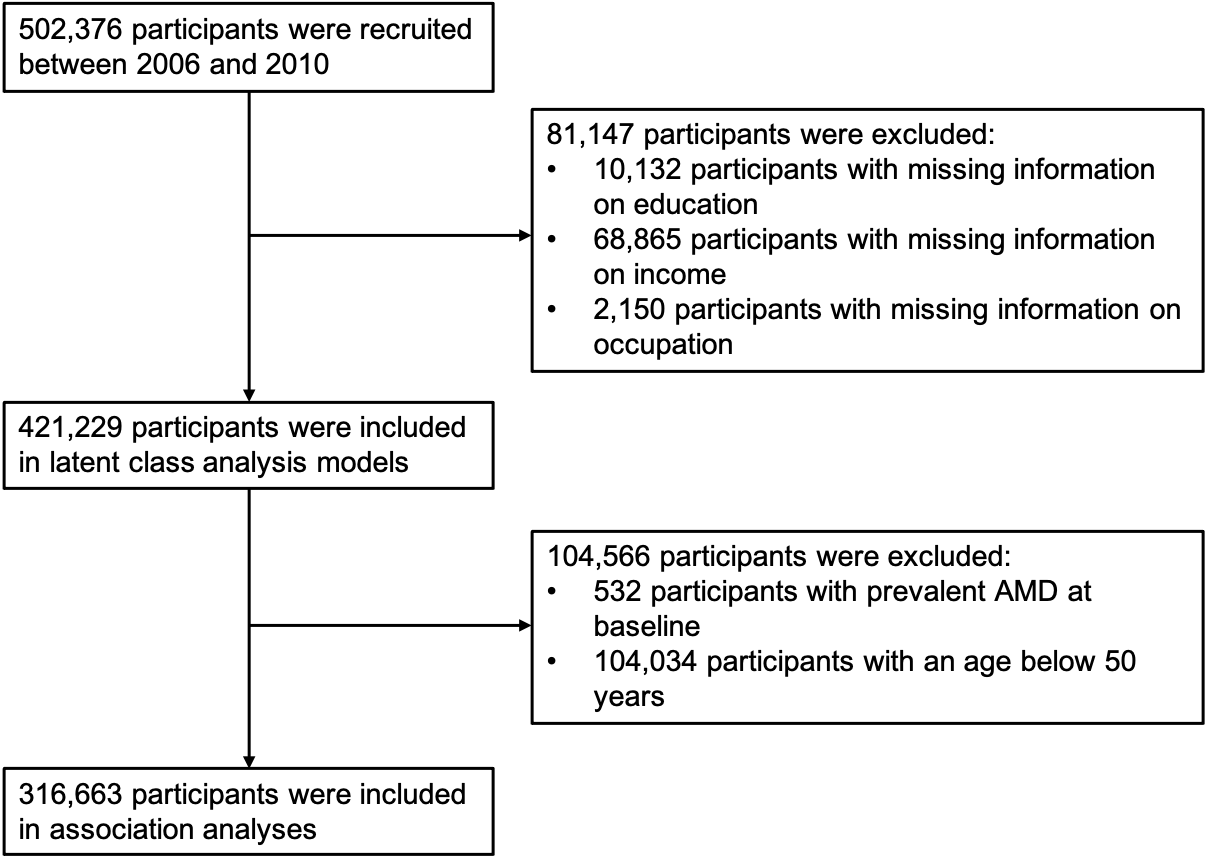


**Figure S1**. Flowchart of the included participants.

**Abbreviations:** AMD, age-related macular degeneration.


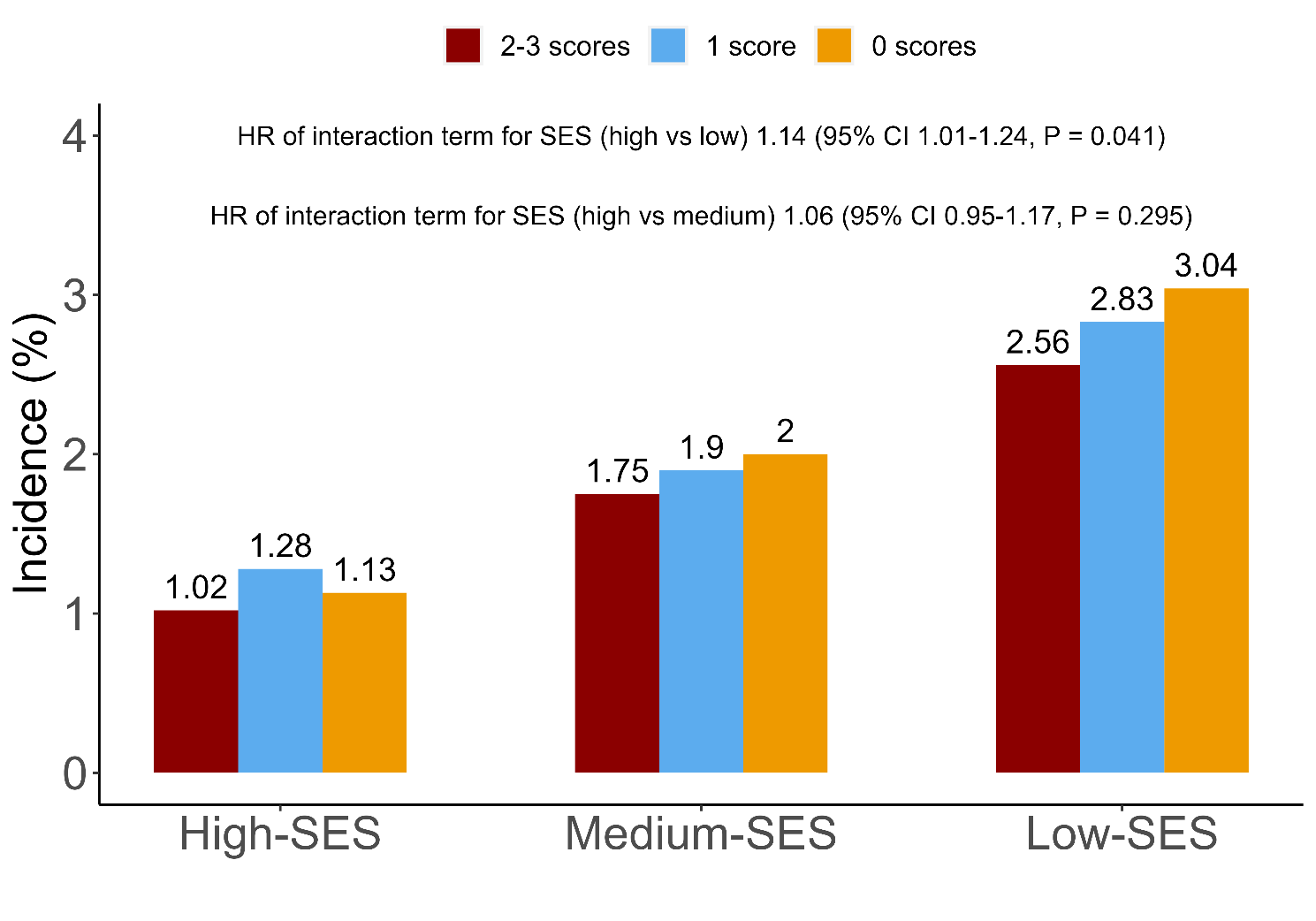


**Figure S2**. Incidence of AMD across different combined categories of SES and lifestyle score.

Multiplicative interaction was evaluated using hazard ratios for the product term between SES and one-point decreasing in the healthy lifestyle score.

Hazard ratios for product term were calculated using Cox proportional hazards analysis after adjusting for baseline age and gender, race, assessment center, BMI, self-report hypertension, self-report diabetes, and self-report cancer.

**Abbreviations:** SES, socioeconomic status; AMD, age-related macular degeneration; HR, hazard ratio; CI, confidence interval.


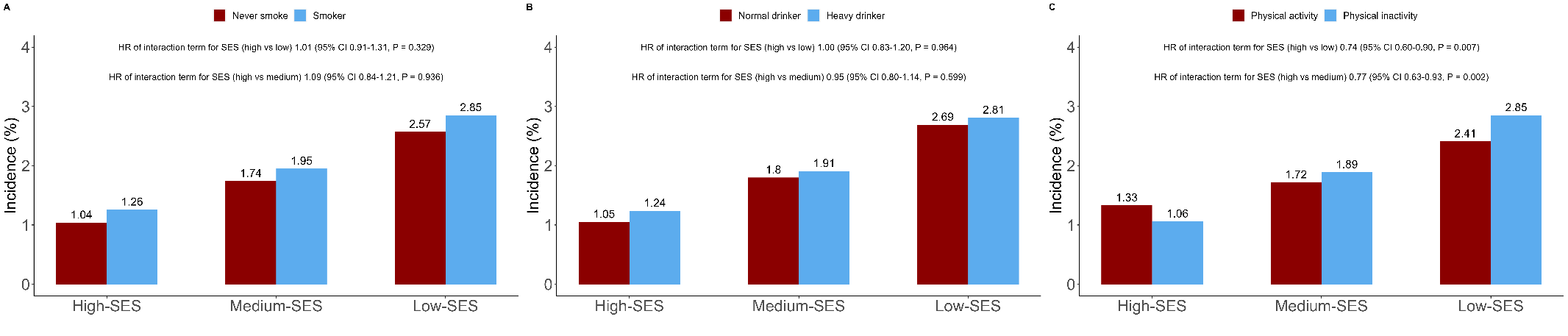


**Figure S3**. Incidence of AMD across different combined categories of SES and each lifestyle factor.

Multiplicative interaction was evaluated using hazard ratios for the product term between bad behaviors and SES.

Hazard ratios for product term were calculated using Cox proportional hazards analyses after adjusting for baseline age and gender, race, assessment center, BMI, self-report hypertension, self-report diabetes, and self-report cancer.

**Abbreviations:** SES, socioeconomic status; AMD, age-related macular degeneration; HR, hazard ratio; CI, confidence interval.


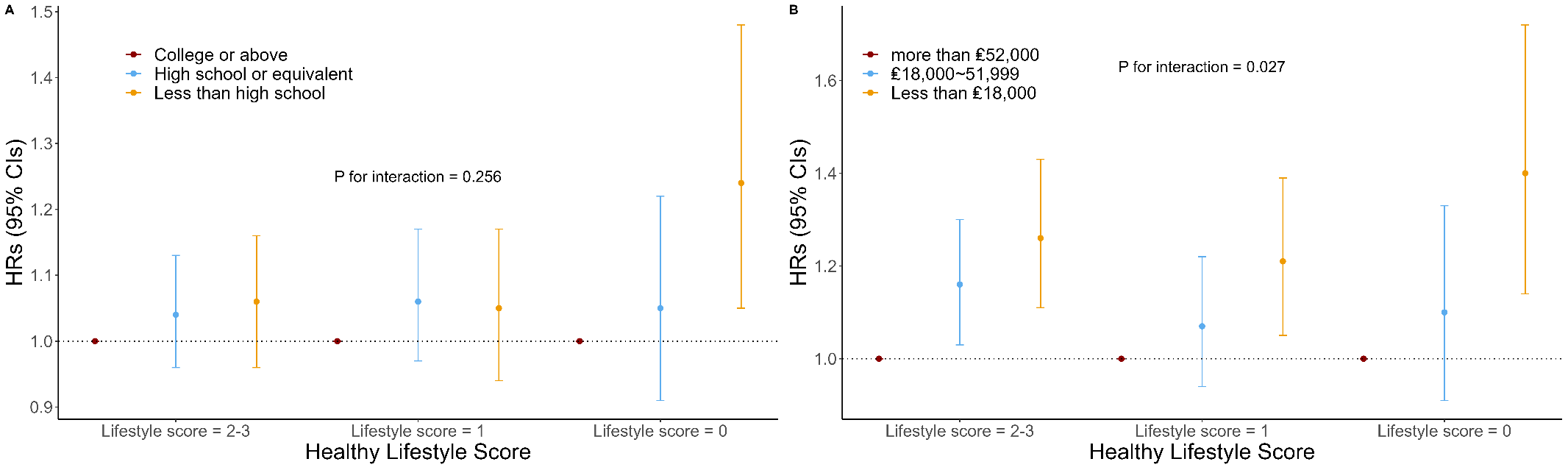


**Figure S4**. Associations of education level and household income and incident AMD by different healthy lifestyle score.

Circles represent hazard ratios; horizontal lines indicate corresponding 95% confidence intervals around hazard ratios. Hazard ratios were calculated using Cox proportional hazards analyses after adjusting for baseline age and gender, race, assessment center, BMI, self-report hypertension, self-report diabetes, and self-report cancer.

**Abbreviations:** SES, socioeconomic status; AMD, age-related macular degeneration; HR, hazard ratio; CI, confidence interval; BMI, body mass index.


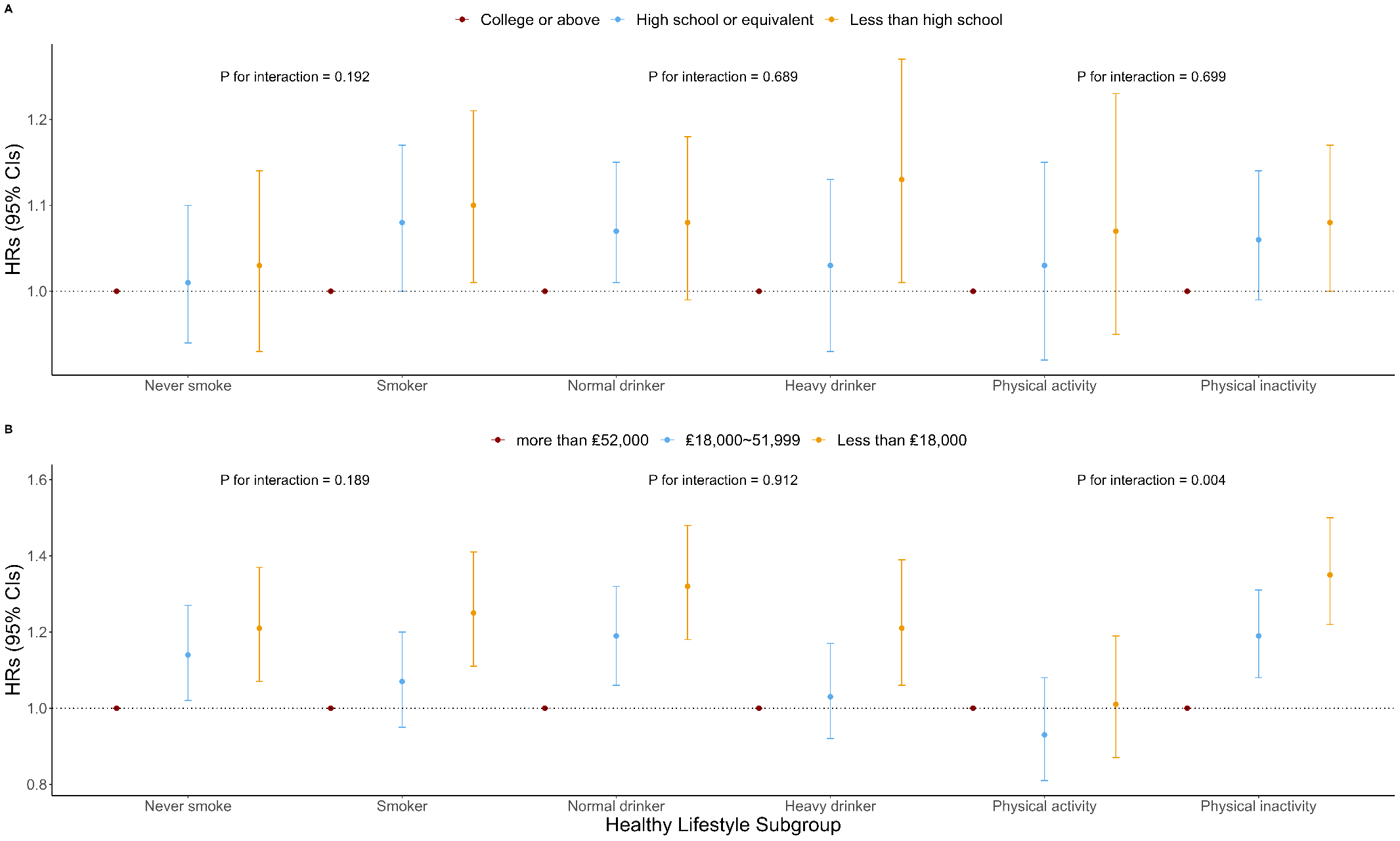


**Figure S5**. Associations of socioeconomic status factors and incident AMD by different lifestyle behavior subgroup.

Circles represent hazard ratios; horizontal lines indicate corresponding 95% confidence intervals around hazard ratios. Hazard ratios were calculated using Cox proportional hazards analyses after adjusting for baseline age and gender, race, assessment center, BMI, self-report hypertension, self-report diabetes, and self-report cancer.

**Abbreviations:** SES, socioeconomic status; AMD, age-related macular degeneration; HR, hazard ratio; CI, confidence interval; BMI, body mass index.


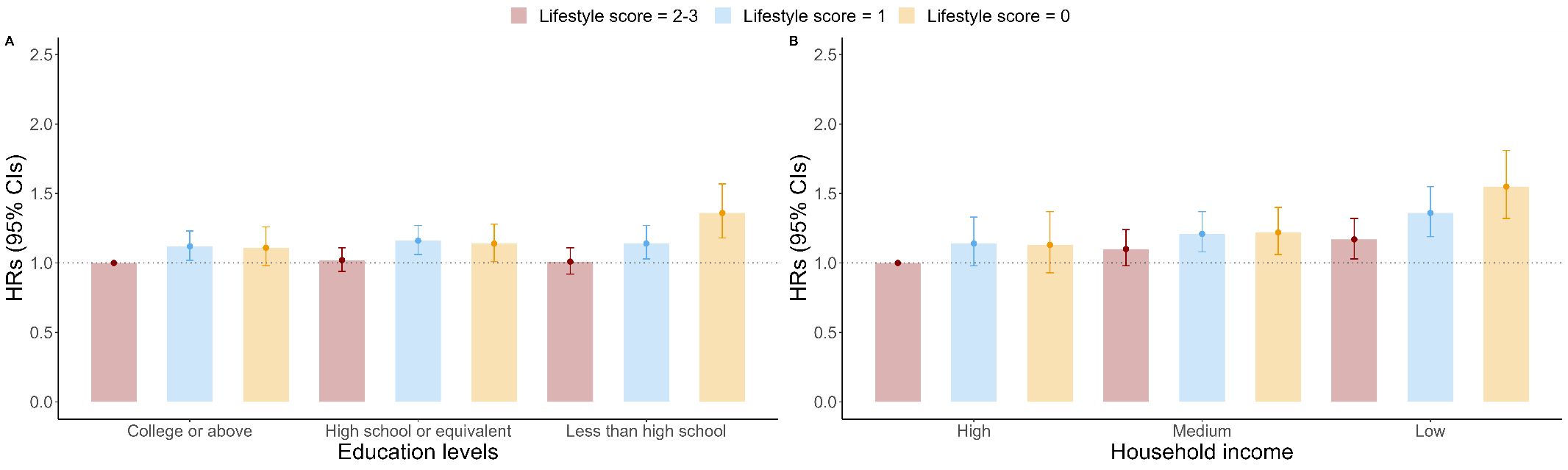


**Figure S6**. Joint associations of healthy lifestyle score and socioeconomic factors with incident AMD.

Circles represent hazard ratios; horizontal lines indicate corresponding 95% confidence intervals around hazard ratios. Hazard ratios were calculated using Cox proportional hazards analyses after adjusting for baseline age and gender, race, assessment center, BMI, self-report hypertension, self-report diabetes, and self-report cancer. Those in the highest SES level group with 2-3 lifestyle score were treated as the reference.

**Abbreviations**: AMD, age-related macular degeneration; SES, socioeconomic status; HR, hazard ratios; CI, confidence intervals; BMI, body mass index.
